# DMRTA2 Regulates Radial Glial Maintenance and Tumorigenicity of Paediatric High‐Grade Glioma

**DOI:** 10.1111/jcmm.71092

**Published:** 2026-03-17

**Authors:** Hitomi N. Royston, Autumn B. Hampton, Elissa G. Oliver, Jayden Jackson, Ibukunoluwa Tella, Miriam D. Emerson, Dhruv Bhagat, Grace E. Zimmerman, Daijiro Konno, Kosuke Funato

**Affiliations:** ^1^ Center for Molecular Medicine University of Georgia Athens Georgia USA; ^2^ Department of Biochemistry and Molecular Biology University of Georgia Athens Georgia USA; ^3^ Institute of Bioinformatics University of Georgia Athens Georgia USA; ^4^ Cellular and Molecular Bioengineering Laboratory, Graduate School of Science and Engineering Kindai University Osaka Japan

## Abstract

DMRTA2 is a member of the evolutionarily conserved transcription factor family involved in various developmental processes including neurogenesis. However, the roles of DMRTA2 in human development and disease are not fully characterised. Single cell RNA‐seq data analysis showed that DMRTA2 is robustly expressed in neural progenitors, in particular radial glial (RG) cells, in the human fetal brain. Knockout of the *DMRTA2* gene in human embryonic stem cell (hESC)‐derived cerebral organoids led to smaller organoid size and fewer RG cells. We also found that DMRTA2 is highly and specifically expressed in diffuse hemispheric gliomas, H3G34‐mutant (DHG‐H3G34), a malignant subtype of paediatric brain tumour. Loss of DMRTA2 resulted in enhanced neuronal differentiation, fewer RG‐like glioma cells and impaired tumorigenicity, suggesting its important role in both normal brain development and the formation of malignant brain tumours.

## 1 Introduction

DMRTA2 (also known as DMRT5) is a member of the DMRT (Doublesex‐mab3‐related transcription factor) family that is characterised by the conserved zinc finger DNA‐binding domain called DM (Doublesex/Mab‐3) domain [1]. The DMRT family genes are involved in various developmental processes, including sex determination and neurogenesis. In the mouse fetal brain, Dmrta2 is highly expressed in the Sox2‐positive neural progenitor cells (NPCs), and *Dmrta2* knockout (KO) mice show abnormal forebrain development, including a 40% reduction in cortical size, loss of hippocampal structure, and reduced proliferation of NPCs [2, 3]. Dmrta2 directly regulates the expression of the *Hes1* gene, which is critical for the maintenance of NPCs [4]. *Dmrta2* KO in mouse embryonic stem cell (ESCs)‐derived NPCs accelerates the cell cycle exit and differentiation into postmitotic neurons. These observations are consistent with a case report that identified *DMRTA2* as a causal gene of hereditary lissencephaly [5]. These findings collectively highlight the important role of DMRTA2 in the proliferation and maintenance of NPCs.

High‐grade glioma (HGG) is one of the deadliest types of cancer affecting both adults and children [6]. Paediatric HGG accounts for 40% of brain tumour deaths in children. Thus, there is an urgent clinical need to understand tumour biology and develop new therapies. It has been shown that paediatric brain tumours are caused by a dysregulated developmental program that maintains cells in an immature state [7]. The close link between brain development and brain tumour formation is also evident from recent studies that showed the presence of radial glia (RG)‐like cells in HGGs [8, 9, 10]. RG is a type of neural progenitor that exists in the fetal brain and is characterised by its unique morphology and gene expression profile, and RG‐like glioma cells molecularly and morphologically resemble RG. Due to their high migratory capacity and cell cycle state, RG‐like glioma cells are implicated in treatment resistance and tumour relapse. However, the molecular mechanism underlying the maintenance of RGs and RG‐like glioma cells is not fully understood. Among various subtypes of paediatric HGGs, diffuse hemispheric gliomas, H3G34‐mutant (DHG‐H3G34) contain RG‐like glioma cells [10]. DHG‐H3G34 primarily affects adolescents and young adults and is characterised by recurrent point mutations in the histone variant H3.3 gene that result in a substitution of the glycine at position 34 by arginine or valine (H3.3G34R/V) [11, 12]. Presently, there is no established therapeutic approach tailored to this subtype, and patients continue to face a grim prognosis with a median survival of only 14.4 months following diagnosis [13].

In this study, we used human embryonic stem cell (hESC)‐based models to investigate the role of DMRTA2 in normal brain development as well as the formation of DHG‐H3G34. We established *DMRTA2* knockout (KO) ESC lines by using CRISPR/Cas9 gene editing and differentiated them to cerebral organoids. *DMRTA2* KO organoids exhibited smaller size, decreased proliferation, and fewer RGs compared to *DMRTA2* wildtype organoids. Furthermore, *DMRTA2* was knocked out in DHG‐H3G34 cells. *DMRTA2* KO resulted in an increase in neuronal differentiation as well as a decrease in tumorigenicity. Taken together, our findings indicate an important role of DMRTA2 in maintaining RG identity and tumorigenicity of paediatric HGGs.

## 2 Materials and Methods

### 2.1 Analysis of Single Cell RNA‐Seq Data

Single cell RNA‐seq datasets were analysed by the Seurat (v5.2.1) package [14] with R (v4.4.1) software using the standard workflow. Briefly, low quality/dead cells were removed based on gene count and mitochondrial gene contamination. After normalisation, the Uniform Manifold Approximation and Projection (UMAP) non‐linear dimensional reduction was performed for 2‐dimensional visualisation.

### 2.2 hESC and Cerebral Organoid Culture

H1 hESCs (NIHhESC‐10‐0043) were obtained from WiCell and maintained in Essential 8 FLEX medium (Thermo Fisher, A2858501) on cell culture dishes coated with recombinant Vitronectin (Thermo Fisher, A14700). Cerebral organoids were derived from hESCs using a previously described protocol [15]. Briefly, hPSCs were dissociated with Accutase (Innovative Cell Technologies), and 10,000 cells were plated into each well in a V‐bottom 96 well plate. The next day, medium was changed to Essential 6 medium (Thermo Fisher, A1516401) with 200 nM of BMP inhibitor LDN193189 (STEMCELL Technologies, 72,149), 10 μM of SMAD inhibitor SB431542 (STEMCELL Technologies, 100–1051), and 2 μM of Wnt inhibitor XAV939 (Cayman Chemical, 13,596). From day 8, organoids were cultured in Neurobasal medium (Gibco, 21,103,049) supplemented with B‐27 supplement w/o vitamin A (Gibco, 12,587,010, 1:100), N2 supplement (Gibco, 17,502,048, 1:200), GlutaMax (Gibco, 35,050,061, 1:100), 0.055 mM cell culture grade 2‐mercaptoethanol solution (Gibco, 21,985,023), 2.5 μg/mL Insulin (MP Biomedicals, 0219390025), and 10 ng/mL Human LIF (Millipore, LIF1010). Medium change was performed every other day, and organoids were moved to an orbital shaker on day 14. The DHG‐H3G34 model cells were prepared as previously described [16]. Mycoplasma contamination and sterility were tested routinely.

### 2.3 Immunohistochemistry

Cells were fixed with 4% paraformaldehyde (PFA), rinsed with PBS, and incubated with a blocking solution containing 10% FBS and 0.3% Triton X‐100 for 1 h. Antibodies, including mouse monoclonal antibody against DLX2 (Santa Cruz, sc‐393,879, 1:200) and rat monoclonal antibody against SOX2 (eBioscience, 14–9811‐82, 1:200), were diluted in the blocking solution and applied to samples overnight at 4°C. Subsequently, samples were washed three times with PBS, incubated with secondary antibodies in PBS with 0.1% bovine serum albumin (BSA), and followed by nuclear staining using DAPI. Cryosections were stained with antibodies against human nuclear antigen (HNA) (Abcam, ab191181, 1:2000), Ki67 (R&D, MAB7617, 1:200), human NCAM (Santa Cruz, sc‐106, 1:400), and SOX2 (eBioscience, 14–9811‐82, 1:200). Haematoxylin and eosin (H&E) staining was performed using Haematoxylin and Eosin Y solutions obtained from MilliporeSigma.

### 2.4 Western Blot

Cells were lysed in RIPA buffer, and after a 30‐min centrifugation at 16,900 rpm, the supernatant was collected. Protein concentration was determined using the Bradford Assay (Bio‐Rad). Lysates were boiled for 5 min in Laemmli sample buffer, separated by SDS‐PAGE, and then transferred to a nitrocellulose or PVDF membrane. The membrane was blocked with a 5% Blotting‐Grade Blocker (Bio‐Rad) in TBS‐T and was incubated at 4°C overnight in the blocking buffer with primary antibodies. Anti‐DMRTA2 rabbit polyclonal antibody (1:2000) was provided by Dr. Daijiro Konno. Anti‐β‐actin (Cell Signaling Technology, 3700, 1:2000) or anti‐β‐tubulin (Santa Cruz, sc‐32,293, 1:2000) was used for loading control. After three washes with TBS‐T, the blot was incubated with the respective secondary antibodies for mouse (1:5000) or rabbit (1:5000) at room temperature for 30 min. Clarity ECL Western Blotting Substrates (Bio‐Rad) were used for detection according to the manufacturer's instructions. For re‐blotting, antibodies were stripped by Restore Western Blot Stripping Buffer (Thermo Fisher).

### 2.5 Flow Cytometry

Cells were dissociated with Accutase (Innovative Cell Technologies) and collected into a 15 mL conical tube. Cells were centrifuged at 1500 rpm for 5 min and were washed with ice‐cold PBS two times. Cells were then co‐stained with anti‐GLAST mouse monoclonal antibody conjugated with PE (Miltenyi Biotec, 130–118‐344) and anti‐PTPRZ1 mouse monoclonal antibody (Santa Cruz, sc‐33,664), followed by anti‐mouse IgM secondary antibody conjugated with Alexa Fluor 633 (Invitrogen). Data were acquired on CytoFLEX flow cytometer (Beckman Coulter, Brea, CA, USA) and analysed by FlowJo software.

### 2.6 Intracranial Transplantation

Intracranial transplantation was performed as previously described [17]. Briefly, 500,000 cells suspended in 2 μL of PBS were injected into the target coordinates (2 mm anterior and 2 mm lateral right from the bregma, 2 mm deep from the skull surface) at a rate of 1 μL per minute. The number of cells was determined based on previous studies [16, 17]. All animal experiments are approved by University of Georgia Institutional Animal Care and Use Committee (Animal Use Protocol# A2024 06–012‐A4).

### 2.7 Statistical Analyses

Data were tested for normality using the Shapiro–Wilk test. For data that were not normally distributed, statistical analyses were performed using the Kruskal‐Wallis test (non‐parametric), followed by the Mann Whitney *U* test for pairwise comparisons. For data that were normally distributed, one‐way ANOVA was performed followed by the Student's *t*‐test for pairwise comparisons.

## 3 Results

### 3.1 DMRTA2 Is Highly Expressed in RGs

**FIGURE 1:**
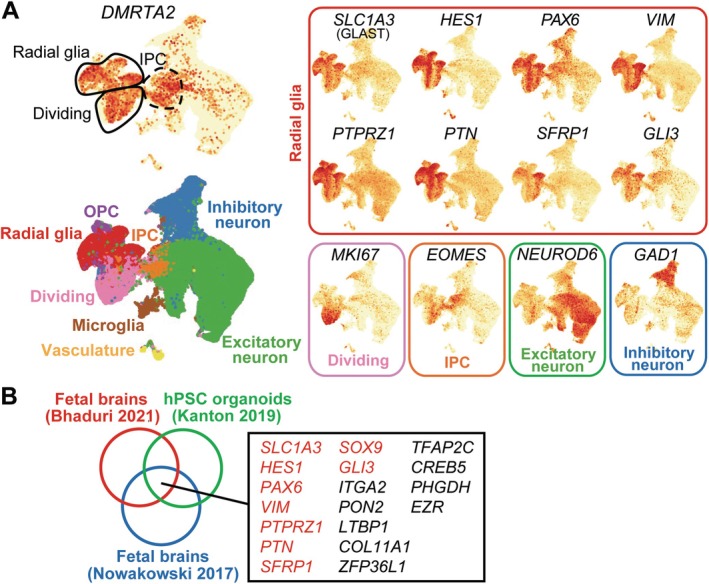
*DMRTA2* is highly expressed in RG cells in the human developing brain. (A) Uniform Manifold Approximation and Projection (UMAP) of single cell RNA‐seq data obtained from human fetal brain [19] shows the expression of *DMRTA2* in RG cells. The expression of lineage marker genes was shown in the right panel. IPC: Intermediate Progenitor Cell, OPC: Oligodendrocyte Progenitor Cell. (B) Genes whose expression is highly correlated with *DMRTA2* expression in all three single cell RNA‐seq datasets are shown. The genes in red font indicate known RG marker genes.

In mouse fetal brain at E10.5, Dmrta2 is robustly expressed in the dorsal telencephalon, mostly in SOX2‐positive neural progenitor cells at the ventricular zone [2]. However, the expression pattern of DMRTA2 in the human developing brain has not been characterised. We thus analysed two single cell RNA‐seq datasets from human fetal brains [18, 19]. *DMRTA2* is highly expressed in RGs and “Dividing” cells, which express Ki67 in addition to RG markers, including *SLC1A3* (also known as GLAST), *HES1* and *PTPRZ1* (Figure 1A, Figure S1A). *DMRTA2* expression is also observed in a subset of intermediate progenitor cells (IPCs) expressing *TBR2* (also known as EOMES). Similarly, another single cell RNA‐seq dataset from hESC‐derived cerebral organoids showed that *DMRTA2* is expressed in RG cells and IPCs [20] (Figure S1B). We next sought genes whose expression was correlated with that of *DMRTA2* and identified 18 genes shared among the three datasets. These include 9 known RG markers, further confirming the robust expression of *DMRTA2* in RG population (Figure 1B).

### 3.2 *DMRTA2* KO Resulted in a Reduction in Organoid Size and RG Population

**FIGURE 2:**
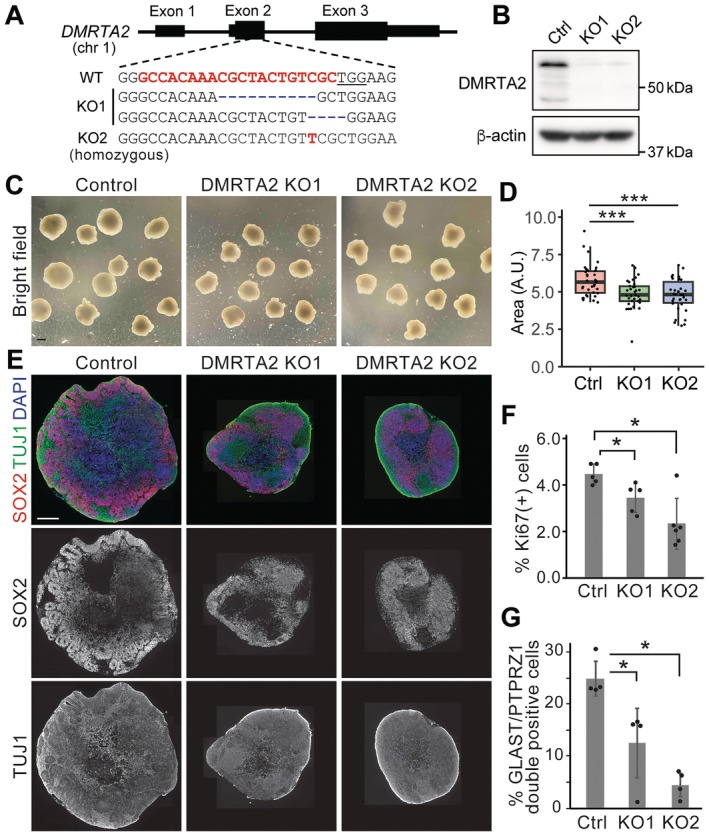
*DMRTA2* KO cerebral organoids exhibit smaller size and fewer RG cells. (A) Schematic illustration depicts mutations in *DMRTA2* KO hESC lines. (B) Western blotting confirmed the loss of DMRTA2 protein in the KO cells. (C) Representative bright field images of cerebral organoids. Scale bar: 1 mm (D) Boxplot shows a reduction of organoid size by *DMRTA2* KO. (33–35 organoids/group). (E) Representative composite images of cerebral organoid sections immunostained with indicated antibodies. Scale bar: 500 μm. (F) Reduction of Ki67‐positive proliferating cells by *DMRTA2* KO. Bars indicate mean ± S.D. (5–6 organoids/group). (G) Flow cytometry showed a decrease of GLAST+PTPRZ1+ double positive RG cells by *DMRTA2* KO. Bars indicate mean ± S.D. (4 independent experiments) *p < 0.05, ***p < 0.001.

To investigate the functional role of DMRTA2 in human brain development, we knocked out the *DMRTA2* gene using CRISPR‐Cas9 genome editing in hESCs and differentiated them into cerebral organoids. Sequencing of genomic DNA and western blotting confirmed frame shift mutations and loss of protein expression, respectively (Figure 2A,B, Figure S2A). Cerebral organoids derived from two *DMRTA2* KO lines exhibited reduced organoid size compared to *DMRTA2* wildtype organoids (Figure 2C,D). Immunofluorescence staining of frozen sections showed that wildtype organoids contained dozens of ventricle‐like structures surrounded by SOX2‐positive neural progenitor cells (Figure 2E). On the other hand, *DMRTA2* KO organoids contained fewer structures, suggesting a defect in RG maintenance. Ki67 staining showed a significant decrease in proliferation by *DMRTA2* KO (Figure 2F, Figure S2B). To evaluate the impact of *DMRTA2* KO on RGs, we dissociated organoids and quantified SLC1A3^+^PTPRZ1^+^ double positive RGs by flow cytometry. *DMRTA2* KO organoids contained a significantly lower number of SLC1A3^+^PTPRZ1^+^ double positive RGs compared to wildtype organoids (Figure 2G, Figure S2C). Taken together, these data indicate the important role of DMRTA2 in cerebral development, especially in the maintenance of RGs.

### 3.3 *DMRTA2* KO Resulted in Fewer RG‐Like Cells in DHG‐H3G34 Cells

**FIGURE 3:**
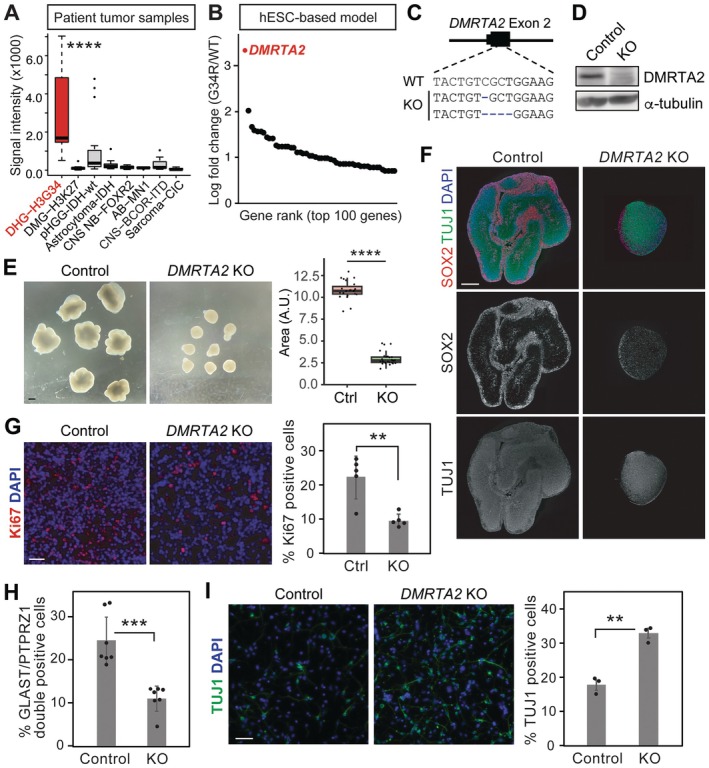
DMRTA2 is required for the maintenance of RG state in DHG‐H3G34 cells. (A) Box plot shows the expression of *DMRTA2* in various subtypes of paediatric brain tumours. (B) RNA‐seq data showed that H3.3G34R mutation upregulates the expression of *DMRTA2*. (C) Frame shift mutations in *DMRTA2* KO DHG‐H3G34 model cells. (D) Western blotting confirmed the loss of DMRTA2 protein in the KO cells. (E) Representative bright field images of organoids derived from DHG‐H3G34 model cells. Scale bar: 1 mm. Boxplot on the right shows a reduction of organoid size by *DMRTA2* KO. (23–28 organoids/group). (F) Representative composite images of organoid sections immunostained with indicated antibodies. Scale bar: 500 μm. (G) Immunostaining showed a reduction of Ki67‐positive proliferating cells by *DMRTA2* KO. Bars indicate mean ± S.D. (5 organoids/group). (H) Flow cytometry showed a decrease of GLAST+PTPRZ1+ double positive RG cells by *DMRTA2* KO. Bars indicate mean ± S.D. (7 independent experiments) (I) Dissociated cells were replated and cultured in neuronal differentiation medium for 14 days. Immunostaining for TUJ1 showed increased neuronal differentiation by *DMRTA2* KO. Scale bar: 100 μm. Bars indicate mean ± S.D. (3 independent experiments) **p < 0.01, ***p < 0.001, ****p < 0.0001.

The gene expression profile of patient tumours obtained from the Gene Expression Omnibus (GEO) database (GSE34824, GSE36245, and GSE73038) showed a robust and specific expression of *DMRTA2* in DHG‐H3G34 compared to other subtypes of paediatric brain tumours (Figure 3A). We also analysed the RNA‐seq data from our previous study [16] and found that *DMRTA2* was the most up‐regulated gene in response to H3.3G34R mutation (Figure 3B), consistent with the finding from another study that showed a downregulation of DMRTA2 expression by H3.3G34R KO [21]. To investigate the role of *DMRTA2* in DHG‐H3G34, we knocked out *DMRTA2* in hESC‐based DHG‐H3G34 model cells in which H3.3G34R, *ATRX* KO, and *TP53* KO were introduced into hESC‐derived ventral forebrain neural progenitors [16] (Figure 3C,D). We confirmed that *DMRTA2 KO* did not interfere with the maintenance of hESC as well as neural induction (Figure S3). Similar to the cortical organoid model, *DMRTA2* KO organoids exhibited significantly smaller organoid size, fewer structures, and reduced proliferation compared to the control organoids (Figure 3E–G). Additionally, flow cytometry analysis showed that *DMRTA2* KO in the DHG‐H3G34 model resulted in a significant decrease in SLC1A3^+^PTPRZ1^+^ double positive RG‐like population (Figure 3H). We next dissociated and replated cells in neuronal differentiation medium. After 2 weeks of culture, neuronal differentiation was evaluated by immunostaining for the post‐mitotic neuronal marker TUJ1. *DMRTA2* KO cells exhibited significantly higher TUJ1 positive cells compared to the DMRTA2 wildtype cells (Figure 3I). These data collectively indicate that DMRTA2 maintains RG‐like state and suppresses neuronal differentiation in DHG‐H3G34 cells.

### 3.4 DMRTA2 Promotes the Tumorigenicity of DHG‐H3G34

**FIGURE 4:**
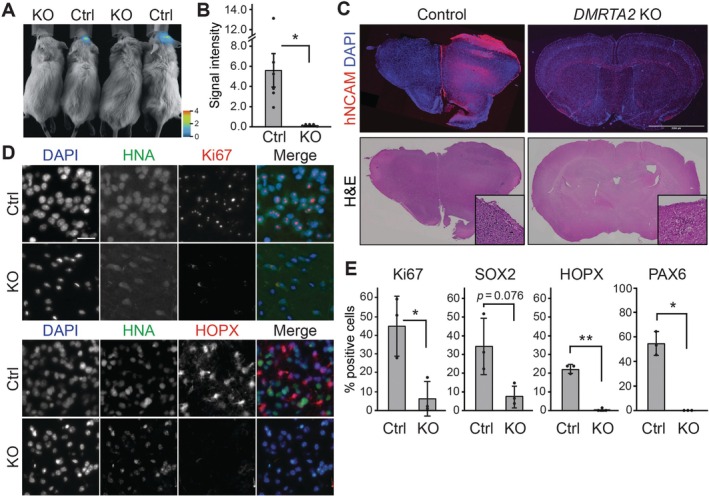
DMRTA2 contributes to the tumorigenicity of DHG‐H3G34. (A) Representative images of in vivo bioluminescence imaging. (B) Quantification of bioluminescence imaging. Bars indicate mean ± S.D. (5–6 mice/group) (C) Representative composite images of brain sections immunostained with anti‐human NCAM antibody (top) or stained with H&E (bottom). Scale bar: 2 mm. (D) Immunostaining for the proliferation marker Ki67, RG marker HOPX, and human nuclear specific antigen (HNA). Scale bar: 50 μm. (E) Quantification of immunostaining. Bars indicate mean ± S.D. (3 mice/group) *p < 0.05, **p < 0.01.

Based on these findings, we next evaluated the role of DMRTA2 in tumorigenicity. *DMRTA2* wildtype and KO DHG‐H3G34 model cells were labelled with the firefly luciferase gene and transplanted into the brains of NOD scid gamma (NSG) immunodeficient mice. In vivo bioluminescence imaging at 6 months post‐transplantation showed significantly lower signals in the KO cohort compared to the control cohort (Figure 4A,B). After sacrifice, the brains were analysed by immunohistochemistry. Immunostaining with a human/primate‐specific anti‐NCAM/CD56 antibody (clone ERIC‐1) showed that *DMRTA2* wildtype cells formed large tumours, while only a small number of human cells were found in the brains of the KO cohort, corroborating our bioluminescence data (Figure 4C). Ki67 staining showed a significant decrease in proliferation by *DMRTA2* KO (Figure 4D,E). Also, the number of SOX2 positive stem/progenitor population was modestly decreased by *DMRTA2* KO, albeit the difference was not statistically significant (*p* = 0.076) (Figure 4E, Figure S4). Additionally, the number of cells that express radial glial markers such as HOPX [18] and PAX6 was significantly decreased by *DMRTA2* KO (Figure 4D,E, Figure S4). Taken together, these results show that DMRTA2 maintains cells in an undifferentiated state, including RG‐like state, and contributes to the tumorigenicity of DHG‐H3G34.

## 4 Discussion

*DMRTA2* and other DMRT family genes play an essential role in brain development, including neural progenitor expansion, lineage specification, and regional patterning [22, 23, 24, 25, 26]. However, most studies were done in mouse or zebrafish models. In this study, we investigated the role of DMRTA2 in human brain development by analysing human single cell RNA‐seq datasets and using a hESC‐based cortical organoid model. Our data demonstrate the robust expression of DMRTA2 in RG cells in the developing brain in independent datasets (Figure 1). The cerebral organoids derived from *DMRTA2* KO hESCs exhibited a smaller size, reduced proliferation, and fewer RG cells (Figure 2). The observed size reduction was smaller than but comparable to that in *Dmrta2* KO mice (40% reduction of the cortical area) [2, 22], indicating an evolutionarily conserved role of DMRTA2.

DHG‐H3G34 is a malignant subtype of paediatric brain tumours. Treatment approach for most of the patients, particularly young adults, closely resembles that of adult glioblastoma, involving a combination of surgery, radiation, and temozolomide‐based chemotherapy [27]. However, patients still face a dismal prognosis with a two‐year overall survival rate at 27.3% [6]. The limited effectiveness of current therapies poses an urgent need to expand our therapeutic options. Our data showed that DMRTA2, which is upregulated by H3.3G34R mutation, is required for maintaining undifferentiated state (Figure 3) and, more importantly, for tumour formation in vivo (Figure 4). Previous studies from us and others showed that DHG‐H3G34 tumours originate from the interneuronal lineage [10, 16, 28]. Intriguingly, DMRTA2 is not normally expressed in the interneuronal lineage. The aberrant expression of DMRTA2 may disrupt the developmental program and maintain the cells in undifferentiated state. A recent study showed that DHG‐H3G34 tumours contain RG‐like cells that transcriptionally resemble RG cells in the developing brain [10]. Normally, RG population diminishes postnatally. The presence of RG‐like cells in DHG‐H3G34 implies that the pathogenic mechanism of DHG‐H3G34 is closely tied to the maintenance of RG cells. Our data obtained from hESC‐based cortical organoid model and DHG‐H3G34 tumour model indicate that DMRTA2 contributes to such mechanism. Given the potential clinical implication of RG‐like glioma cells [8, 9], a better understanding of the maintenance mechanism will pave the way for the development of new therapeutic and diagnostic approaches.

A recent study showed the oncogenic role of DMRTA2 in adult glioblastoma, the most aggressive type of glioma [29]. The expression of DMRTA2 is higher in glioblastoma than that in lower grade glioma, and DMRTA2 knock‐down in patient‐derived cell lines resulted in decreased sphere‐forming capacity. Additionally, vascular net formation of endothelial cells was disrupted by DMRTA2 knock‐down, indicating a potential role of DMRTA2 in tumour angiogenesis. These findings indicate that DMRTA2 plays an important role not only in DHG‐H3G34 but also adult glioblastoma. Further investigation is required to fully elucidate the underlying molecular mechanisms and develop therapies that directly or indirectly target DMRTA2.

## Author Contributions

**Kosuke Funato:** conceptualisation, investigation, formal analysis, supervision, funding acquisition, writing – original draft, writing – review and editing. **Hitomi N. Royston:** investigation, formal analysis, resources, writing – original draft, writing – review and editing. **Autumn B. Hampton, Elissa G. Oliver, Jayden Jackson, Ibukunoluwa Tella, Dhruv Bhagat, Grace E. Zimmerman:** investigation, writing – review and editing. **Miriam D. Emerson:** investigation, resources, writing – review and editing. **Daijiro Konno:** resources, writing – review and editing.

## Funding

This work was supported by the National Institute of Neurological Disorders and Stroke (Grant R01NS131659).

## Conflicts of Interest

The authors declare no conflicts of interest.

## Supporting information


**Figure S1:** *DMRTA2* expression in human RG cells. (A) Single cell RNA‐seq data from human developing brain [18] showed the expression of *DMRTA2* in radial glia (RG) and Intermediate Progenitor Cells (IPCs). The expression of lineage marker genes was shown in the right panel. (B) Single cell RNA‐seq data from human cerebral organoids [20] showed the expression of *DMRTA2* in RG cells and IPCs.
**Figure S2:** DMRTA2 KO in human cerebral organoids. (A) Sanger sequencing of genomic DNA confirmed the KO of *DMRTA2*. (B) Immunostaining showed reduced proliferation by *DMRTA2* KO. Scale bar: 100 μm. (C) Representative 2D plots show gating strategy of the flow cytometry analysis.
**Figure S3:** DMRTA2 KO in DHG‐H3G34 model cells. Immunostaining showed the expression of the neuronal marker SOX2 and interneuronal marker DLX2 in *DMRTA2* KO cells. Scale bar: 50 μm.
**Figure S4:** Immunostaining of DMRTA2 KO xenograft. Immunostaining for the neural stem cell marker SOX2, RG marker PAX6, and human nuclear specific antigen (HNA). Scale bar: 50 μm.

## Data Availability

Data were deposited in Figshare.com (DOI: 10.6084/m9.figshare.29497694). All unique reagents generated in this study are available from the corresponding author upon request.
