# Supplementary figures and images for: DMRTA2 Regulates Radial Glial Maintenance and Tumorigenicity of Paediatric High‐Grade Glioma
Source: J Cell Mol Med. 2026 Mar 17;30(6):e71092. doi: 10.1111/jcmm.71092 (PMC13098098; doi:10.1111/jcmm.71092)

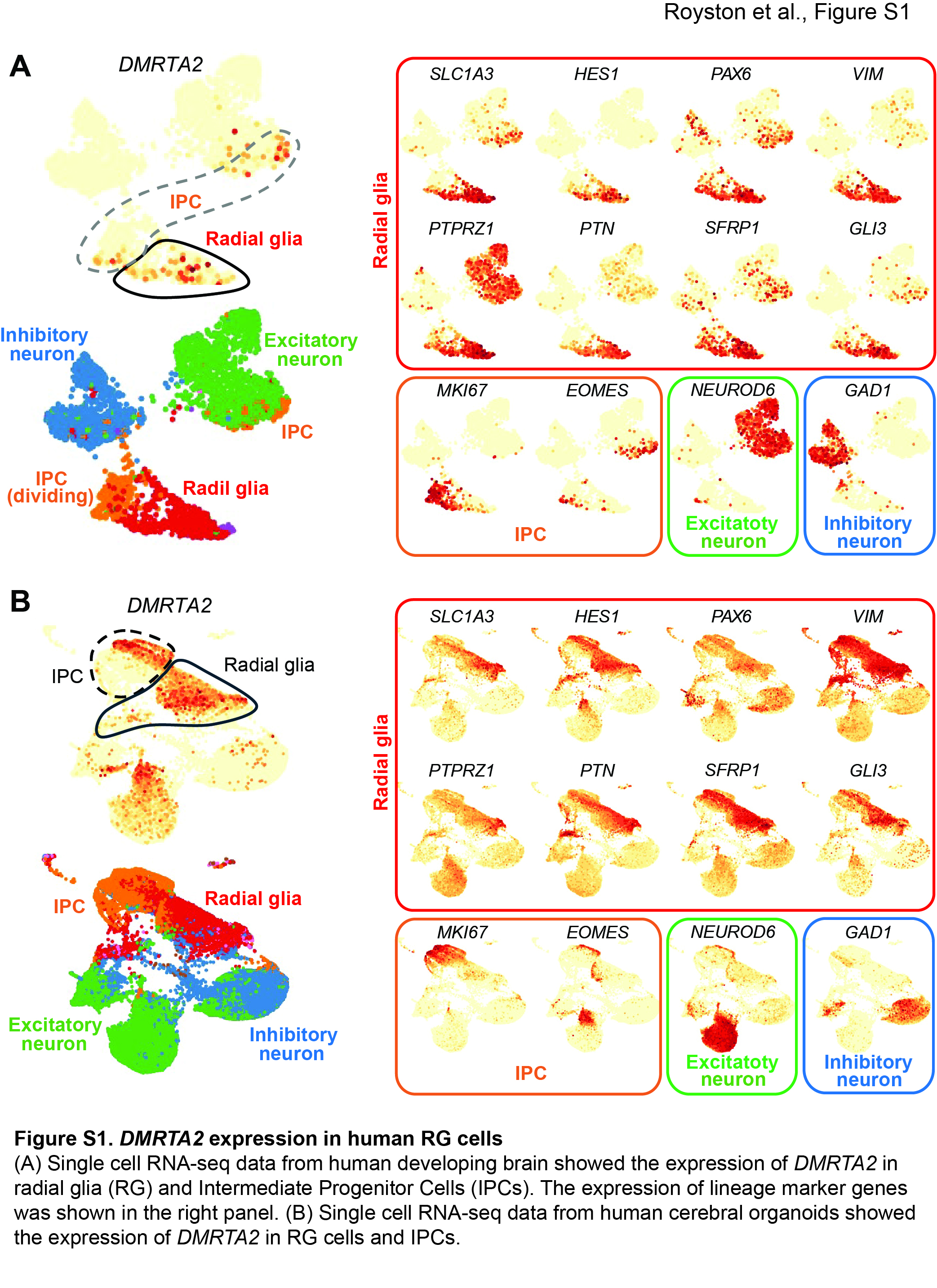

Supplement: Supplementary file 1 — Figure S1: DMRTA2 expression in human RG cells. (A) Single cell RNA‐seq data from human developing brain [18] showed the expression of DMRTA2 in radial glia (RG) and Intermediate Progenitor Cells (IPCs). The expression of lineage marker genes was shown in the right panel. (B) Single cell RNA‐seq data from human cerebral organoids [20] showed the expression of DMRTA2 in RG cells and IPCs. Figure S2: DMRTA2 KO in human cerebral organoids. (A) Sanger sequencing of genomic DNA confirmed the KO of DMRTA2. (B) Immunostaining showed reduced proliferation by DMRTA2 KO. Scale bar: 100 μm. (C) Representative 2D plots show gating strategy of the flow cytometry analysis. Figure S3: DMRTA2 KO in DHG‐H3G34 model cells. Immunostaining showed the expression of the neuronal marker SOX2 and interneuronal marker DLX2 in DMRTA2 KO cells. Scale bar: 50 μm. Figure S4: Immunostaining of DMRTA2 KO xenograft. Immunostaining for the neural stem cell marker SOX2, RG marker PAX6, and human nuclear specific antigen (HNA). Scale bar: 50 μm. [file JCMM-30-e71092-s001.zip › old/jcmm71092-sup-0001-FigureS1@DMRTA2_FigS1.jpg]

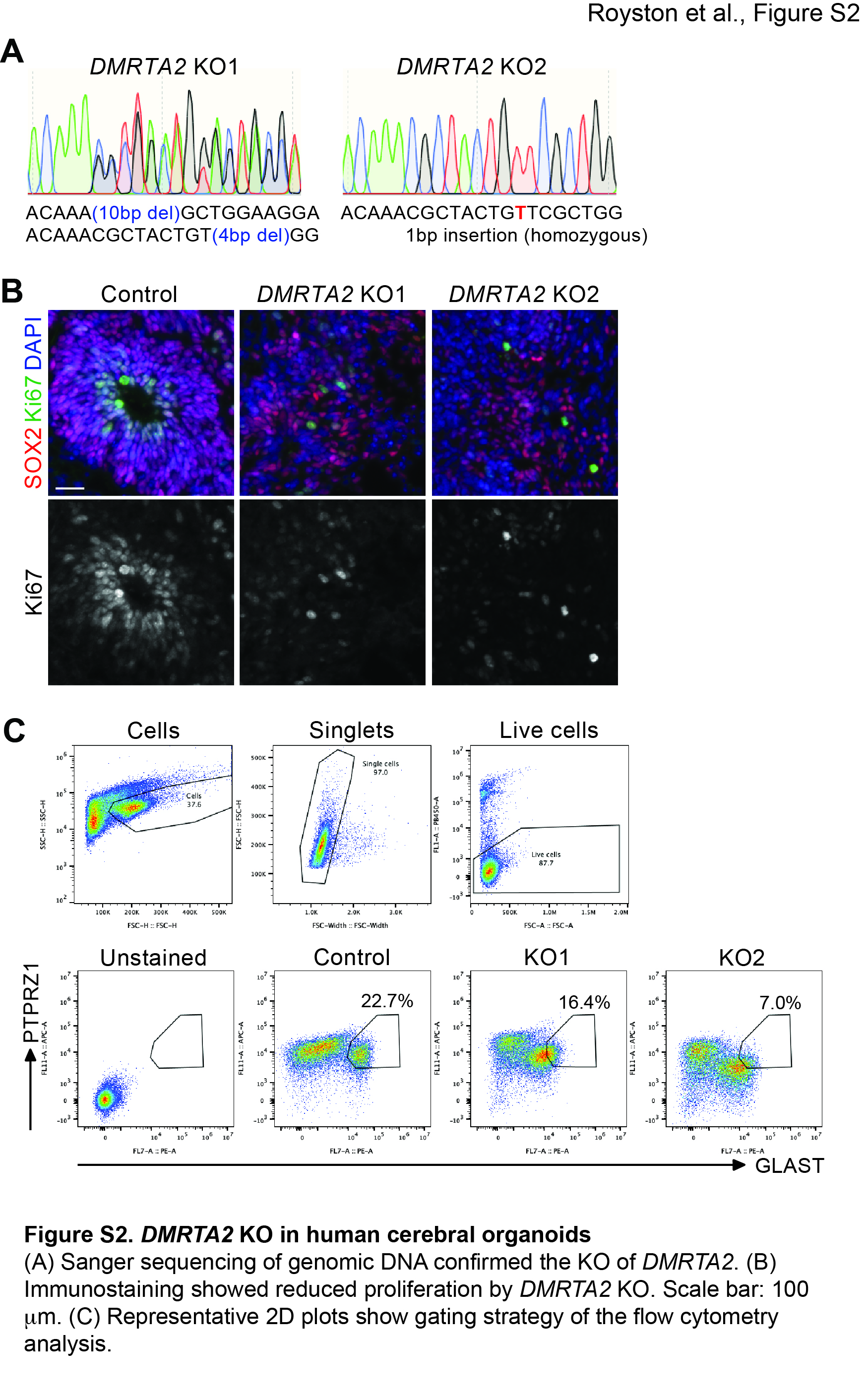

Supplement: Supplementary file 1 — Figure S1: DMRTA2 expression in human RG cells. (A) Single cell RNA‐seq data from human developing brain [18] showed the expression of DMRTA2 in radial glia (RG) and Intermediate Progenitor Cells (IPCs). The expression of lineage marker genes was shown in the right panel. (B) Single cell RNA‐seq data from human cerebral organoids [20] showed the expression of DMRTA2 in RG cells and IPCs. Figure S2: DMRTA2 KO in human cerebral organoids. (A) Sanger sequencing of genomic DNA confirmed the KO of DMRTA2. (B) Immunostaining showed reduced proliferation by DMRTA2 KO. Scale bar: 100 μm. (C) Representative 2D plots show gating strategy of the flow cytometry analysis. Figure S3: DMRTA2 KO in DHG‐H3G34 model cells. Immunostaining showed the expression of the neuronal marker SOX2 and interneuronal marker DLX2 in DMRTA2 KO cells. Scale bar: 50 μm. Figure S4: Immunostaining of DMRTA2 KO xenograft. Immunostaining for the neural stem cell marker SOX2, RG marker PAX6, and human nuclear specific antigen (HNA). Scale bar: 50 μm. [file JCMM-30-e71092-s001.zip › old/jcmm71092-sup-0002-FigureS2@DMRTA2_FigS2.jpg]

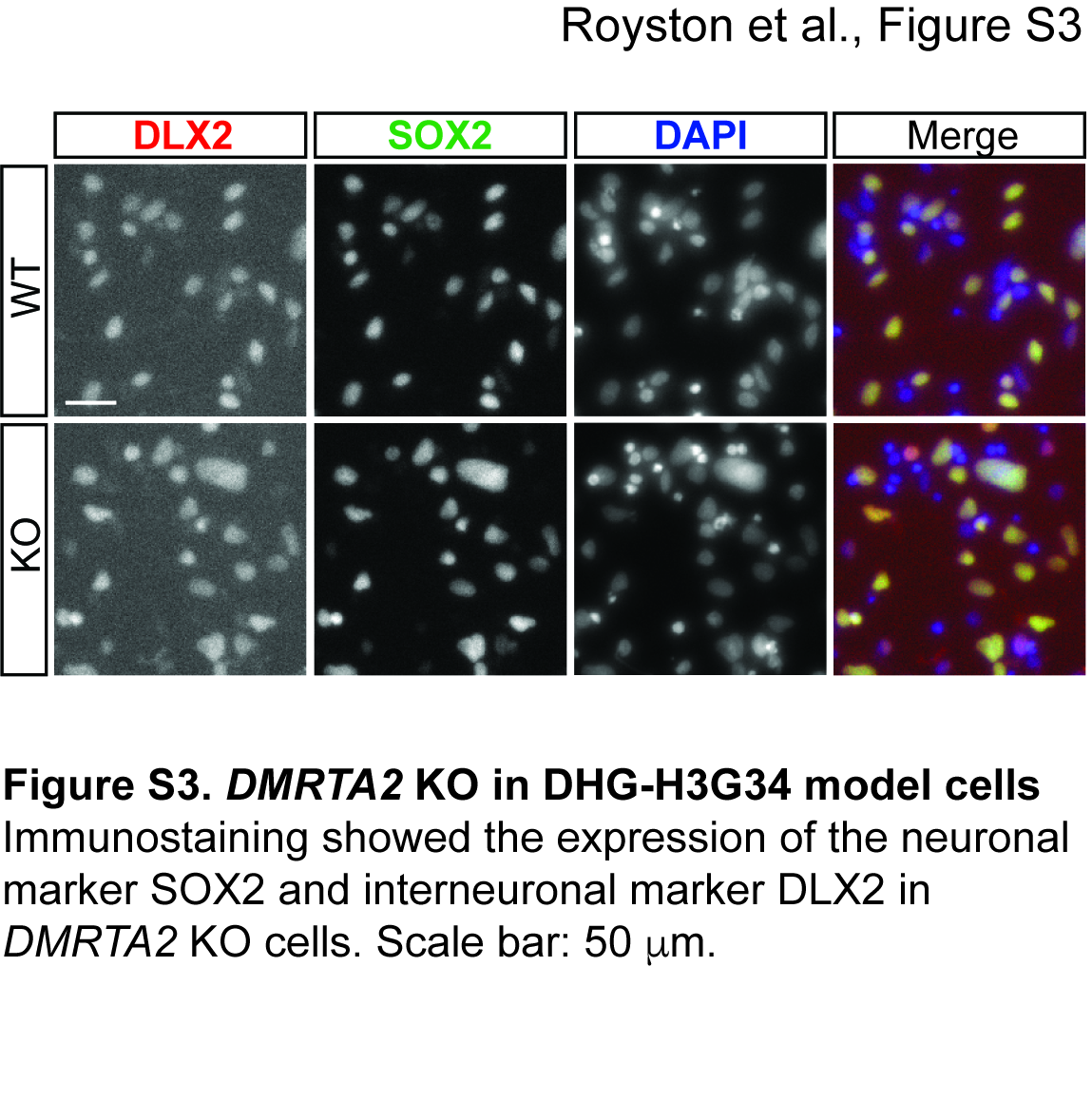

Supplement: Supplementary file 1 — Figure S1: DMRTA2 expression in human RG cells. (A) Single cell RNA‐seq data from human developing brain [18] showed the expression of DMRTA2 in radial glia (RG) and Intermediate Progenitor Cells (IPCs). The expression of lineage marker genes was shown in the right panel. (B) Single cell RNA‐seq data from human cerebral organoids [20] showed the expression of DMRTA2 in RG cells and IPCs. Figure S2: DMRTA2 KO in human cerebral organoids. (A) Sanger sequencing of genomic DNA confirmed the KO of DMRTA2. (B) Immunostaining showed reduced proliferation by DMRTA2 KO. Scale bar: 100 μm. (C) Representative 2D plots show gating strategy of the flow cytometry analysis. Figure S3: DMRTA2 KO in DHG‐H3G34 model cells. Immunostaining showed the expression of the neuronal marker SOX2 and interneuronal marker DLX2 in DMRTA2 KO cells. Scale bar: 50 μm. Figure S4: Immunostaining of DMRTA2 KO xenograft. Immunostaining for the neural stem cell marker SOX2, RG marker PAX6, and human nuclear specific antigen (HNA). Scale bar: 50 μm. [file JCMM-30-e71092-s001.zip › old/jcmm71092-sup-0003-FigureS3@DMRTA2_FigS3.tif]

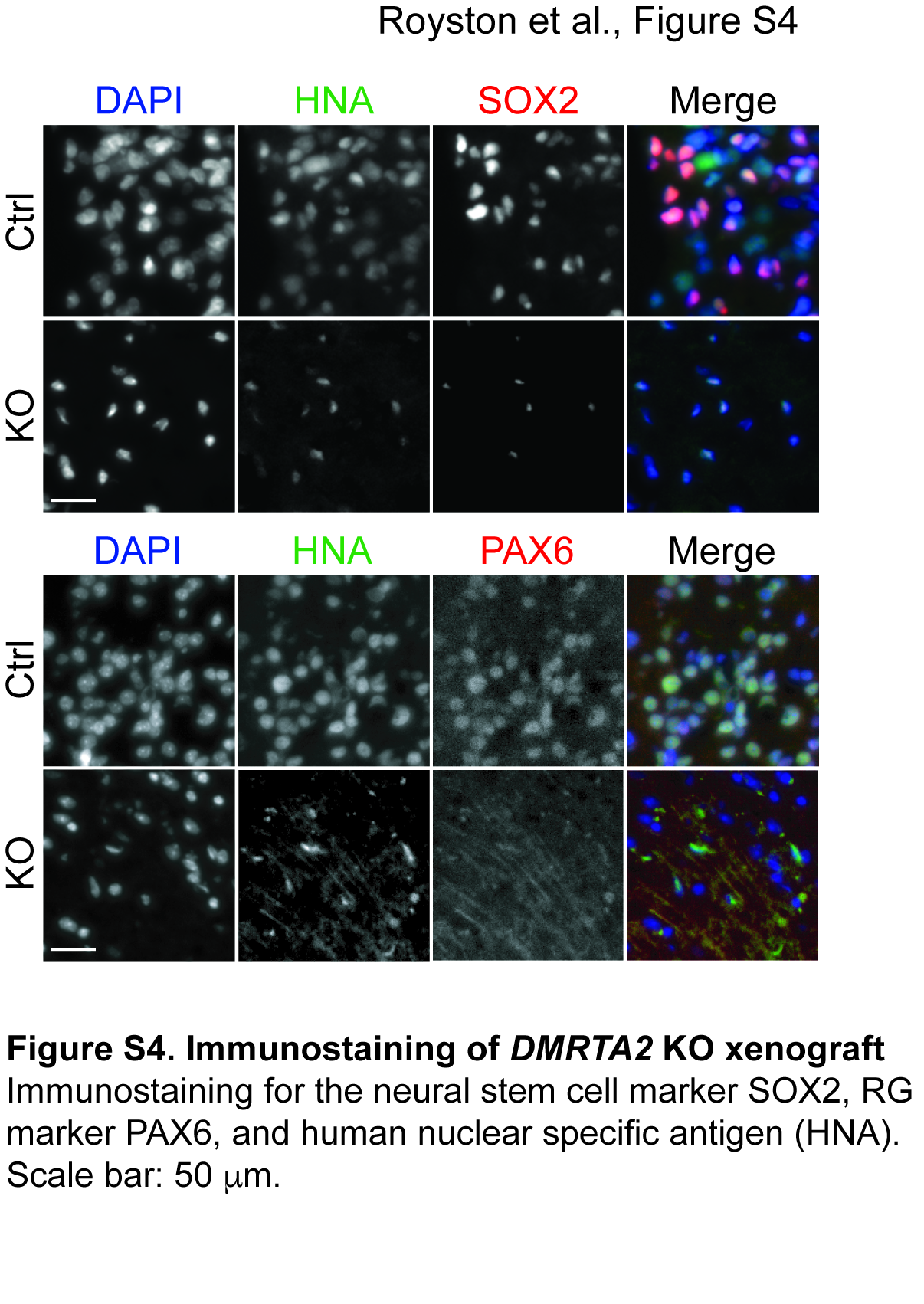

Supplement: Supplementary file 1 — Figure S1: DMRTA2 expression in human RG cells. (A) Single cell RNA‐seq data from human developing brain [18] showed the expression of DMRTA2 in radial glia (RG) and Intermediate Progenitor Cells (IPCs). The expression of lineage marker genes was shown in the right panel. (B) Single cell RNA‐seq data from human cerebral organoids [20] showed the expression of DMRTA2 in RG cells and IPCs. Figure S2: DMRTA2 KO in human cerebral organoids. (A) Sanger sequencing of genomic DNA confirmed the KO of DMRTA2. (B) Immunostaining showed reduced proliferation by DMRTA2 KO. Scale bar: 100 μm. (C) Representative 2D plots show gating strategy of the flow cytometry analysis. Figure S3: DMRTA2 KO in DHG‐H3G34 model cells. Immunostaining showed the expression of the neuronal marker SOX2 and interneuronal marker DLX2 in DMRTA2 KO cells. Scale bar: 50 μm. Figure S4: Immunostaining of DMRTA2 KO xenograft. Immunostaining for the neural stem cell marker SOX2, RG marker PAX6, and human nuclear specific antigen (HNA). Scale bar: 50 μm. [file JCMM-30-e71092-s001.zip › old/jcmm71092-sup-0004-FigureS4@DMRTA2_FigS4.tif]
